# Cognitive outcomes in patients treated with neuromuscular electrical stimulation after coronary artery bypass grafting

**DOI:** 10.3389/fneur.2023.1209905

**Published:** 2023-08-25

**Authors:** Vincenzina Lo Re, Giovanna Russelli, Emanuele Lo Gerfo, Rossella Alduino, Matteo Bulati, Gioacchin Iannolo, Danilo Terzo, Gennaro Martucci, Stefano Anzani, Giovanna Panarello, Gianvincenzo Sparacia, Giuseppe Parla, Federica Avorio, Giuseppe Raffa, Michele Pilato, Aurelio Speciale, Valentina Agnese, Giuseppe Mamone, Fabio Tuzzolino, Giovan Battista Vizzini, Pier Giulio Conaldi, Fabrisia Ambrosio

**Affiliations:** ^1^Neurology Service, Department of Diagnostic and Therapeutic Services, IRCCS ISMETT (Istituto Mediterraneo per i Trapianti e Terapie ad Alta Specializzazione), University of Pittsburgh Medical Center (UPMC), Palermo, Italy; ^2^Department of Research, IRCCS ISMETT, UPMC, Palermo, Italy; ^3^Rehabilitation Service, IRCCS ISMETT, Palermo, Italy; ^4^Department of Anesthesiology and Intensive Care, IRCCS ISMETT, UPMC, Palermo, Italy; ^5^Radiology Unit, Department of Diagnostic and Therapeutic Services, IRCCS ISMETT, Palermo, Italy; ^6^Cardiac Surgery Unit, Department for the Treatment and Study of Cardiothoracic Diseases and Cardiothoracic Transplantation, IRCCS ISMETT, Palermo, Italy; ^7^Salvator Mundi International Hospital, UPMC, Rome, Italy; ^8^Discovery Center for Musculoskeletal Recovery, Schoen Adams Research Institute at Spaulding, Boston, MA, United States; ^9^Department of Physical Medicine and Rehabilitation, Spaulding Rehabilitation Hospital, Charlestown, MA, United States; ^10^Department of Physical Medicine and Rehabilitation, Harvard Medical School, Boston, MA, United States

**Keywords:** post-operative cognitive decline (POCD), neuromuscular electrical stimulation (NMES), coronary artery bypass grafting (CABG), myokines, klotho

## Abstract

**Objective:**

Mechanisms of neurocognitive injury as post-operative sequelae of coronary artery bypass grafting (CABG) are not understood. The systemic inflammatory response to surgical stress causes skeletal muscle impairment, and this is also worsened by immobility. Since evidence supports a link between muscle vitality and neuroprotection, there is a need to understand the mechanisms by which promotion of muscle activity counteracts the deleterious effects of surgery on long-term cognition.

**Methods:**

We performed a clinical trial to test the hypothesis that adding neuromuscular electrical stimulation (NMES) to standard rehabilitation care in post-CABG patients promotes the maintenance of skeletal muscle strength and the expression of circulating neuroprotective myokines.

**Results:**

We did not find higher serum levels of neuroprotective myokines, except for interleukin-6, nor better long-term cognitive performance in our intervention group. However, a greater increase in functional connectivity at brain magnetic resonance was seen between seed regions within the default mode, frontoparietal, salience, and sensorimotor networks in the NMES group. Regardless of the treatment protocol, patients with a Klotho increase 3 months after hospital discharge compared to baseline Klotho values showed better scores in delayed memory tests.

**Significance:**

We confirm the potential neuroprotective effect of Klotho in a clinical setting and for the first time post-CABG.

## Introduction

Cognitive decline is still a common neurologic complication following coronary artery bypass grafting (CABG). Post-CABG cognitive decline affects 43% of patients acutely; this reduces to 19% at 4–6 months but then increases again to 25% of patients between 6 months to 1 year post-operatively (1). The mechanisms of neurocognitive injury are not fully understood. It is known that the systemic inflammation induced by surgery causes blood–brain barrier (BBB) dysfunction, resulting in an influx of inflammatory mediators (2). The increased release of pro-inflammatory cytokines also causes muscle proteolysis, resulting in the loss of skeletal muscle mass. Furthermore, skeletal muscle protein synthesis decreases after surgery since amino acids are directed toward acute phase protein production (3). Altered protein metabolism occurs within 48 h of surgery, the phase when patients have mobility restrictions from being in the intensive care unit (ICU) (4). Bedrest, in turn, exacerbates muscle impairment. Skeletal muscle wasting triggers a devastating cascade of decline in both physical and cognitive functioning. In addition to the critical role of skeletal muscle function in physical mobility, increased attention is being paid to the endocrine function of skeletal muscle as an important secretor of circulating factors (myokines) that play a role in a myriad of systemic functions, including cardiovascular and cognitive health (5, 6). Sarcopenia has been reported to be associated with cognitive decline caused by imbalanced myokine secretion, which leads to upregulation of pro-inflammatory cytokines responsible for memory dysfunction (7). The positive role of physical exercise on brain health suggests that muscle-brain crosstalk, at least in part, may be mediated by myokine signaling. Indeed, exercise promotes hippocampal neurogenesis and blood flow to this part of the brain and, more in general, an improvement in multidomain cognition [reviewed in Severinsen et al. (8)]. Potential activity-induced candidate myokines that have been associated with cognitive function include: Klotho and brain-derived neurotrophic factor (BDNF), which regulate neuronal survival and growth and protect from cardiovascular or cognitive impairment (9–12); fibroblast growth factor 23 (FGF23) and Klotho co-receptor, which may impact brain function, resulting in hippocampal-dependent cognitive impairment if lacking (13–15); and interleukin-6 (IL-6), a pro-inflammatory marker involved in neuroinflammation, which during exercise has an anti-inflammatory effect (5, 16–18). We found this set of physical activity-induced biomarkers interesting because of their differentiated neuroprotective activities: neurogenesis (Klotho, BDNF and FGF-23) and anti-inflammatory effects (IL-6).

In light of this and other evidence supporting a link between muscle vitality and cognitive function, there is a need to preserve skeletal muscle mass and strength during hospitalization for surgical procedures such as CABG. Early mobilization after surgery has multiple benefits for CABG patients, although there is no consensus on the best type, intensity, or duration of rehabilitation (19). Soon after surgery, patients often have no ability to produce effective voluntary muscle contraction because of post-operative pain, hemodynamic instability, the need for mechanical ventilation, sedation, analgesic treatment, and/or tube and catheter placement. For all these reasons, early standard rehab in patients admitted to ICU or semi-intensive units after cardiac surgery is challenging. Conversely, neuromuscular electrical stimulation (NMES) enhances muscle activity without requiring active effort. NMES has also been recognized as a promising tool in cardiovascular rehabilitation (4).

Iwatsu et al. reported the safe use of NMES soon after cardiac surgery (20). Later in the course of recovery, NMES may even be used to attenuate muscle proteolysis and strength loss after cardiac surgery (21). However, data on the post-operative effects of NMES in cardiac surgery patients are conflicting. A randomized clinical trial conducted by Fontes Cerqueira et al. (4) reported that the use of NMES had no effect on walking ability, strength, quality of life or functional outcome in the post-operative period for patients who underwent regular valve replacement. Similarly, Kitamura et al. demonstrated no significant effects of NMES on muscle proteolysis or physical function after cardiovascular surgery (22).

The aim of the present single-center pilot clinical trial was to test our central hypothesis that the addition of an NMES protocol combined with usual care can preserve and enhance skeletal muscle mass in CABG patients. Moreover, we aimed to verify whether the enhanced muscle activity induced by NMES promotes the secretion of myokines important for the preservation of cognitive outcomes.

## Materials and methods

### Study design

A randomized, 2-arm, controlled trial was performed at a single center between February 1st 2019 and July 31st 2020. This study was approved by the Institutional Research Review Board (approval number: 32/17) and the Ethical Committee. Written informed consent was obtained from each participant. Immediately after CABG surgery, adults (≥ 18 years old) were enrolled in the study and randomly assigned to the intervention (NMES + standard rehab) or control group (standard rehab). Randomization was performed using the electronic randomization system provided by RedCap,^1^ the same web application used to collect and analyze the study data. Investigators involved in all assessments were blinded to group assignment.

### Surgical procedure

CABG was performed under general anesthesia. Cardiopulmonary bypass was established through the cannulation of the ascending aorta and the right atrium after full heparin administration. Myocardial protection was achieved by warm blood cardioplegia delivered every 15 min during aortic cross-clamp.

### Participants

Adult patients of both sexes affected with critical coronary artery disease (CAD) and scheduled for elective or urgent CABG surgery were selected if they had been hospitalized for less than 6 days prior to the enrolment date. Participants were recruited during their ICU stay or once they had been transferred to the cardiothoracic unit (CTU), as soon as possible after surgery, and after having signed the informed consent form. Study exclusion criteria were: current use of neuromuscular blocking drugs or vasopressors; fraction of inspired oxygen ≥60%, or positive end-expiratory pressure ≥ 10 cm H_2_0; severe respiratory failure or neurologic disorder with lower extremity function or cognition impairment; blindness; deafness; illiteracy; history of quadriceps or patellar tendon rupture; skin lesions that precluded the application of NMES surface electrodes; current lower extremity fractures; and prisoners. Cognitive and motor impairment was identified by reviewing patients’ medical charts or interviewing a family member when available. Moreover, in order to exclude pre-existing cognitive impairment, a Brief Intelligence Test (TIB) was administered.

### Interventions

For both the NMES and usual care groups, 30-min sessions were conducted twice daily from the enrolment date for 15 days maximum, starting as soon as possible after surgery and continuing during the hospital stay.

#### Usual care group

Subjects received standard care, which included passive mobilization of the limbs, active assisted and/or active exercises (with or without resistance, according to strength level), and cycle ergometry, according to the patient’s consciousness and compliance. Usually, the cycle ergometry setup was as follows: 18–20 revolutions per minute (rpm), resistance 1 or 2 for 30–40 min. When feasible, further rehabilitation included chair sitting and progressive gait training. Intensity of physical activity was decided after a multidisciplinary group discussion and considering clinical status.

#### NMES group

In addition to usual care, subjects in this group completed a low-intensity electrical stimulation protocol to the quadriceps, tibialis anterior (TA), and gastrocnemius muscles bilaterally using the electra stimulator (physioled) (23). Stimulation began on the day of enrolment following baseline assessment. Surface electrode placement and NMES device set-up were performed daily by a trained physical therapist who also checked periodically for skin irritation during the initiation of the NMES protocol. Muscles were stimulated at a pulse frequency of 50 hertz (Hz), with a pulse width of 200 microseconds (μs). The alternating stimulation protocol consisted of a 12-s stimulation of bilateral lower extremity muscles, followed by a 6-s rest, with a 1.6 s ramp-up time and a 1.6 s ramp-down time (24). The intensity of stimulation was gradually increased until a slight muscle contraction was observed; then it was decreased to just subcontractile. This protocol has already been safely used in patients in ICU with no reported major complications (24). Heart rate (HR), systolic blood pressure (SBP), diastolic blood pressure (DBP), saturation of peripheral oxygen (SpO_2_), and respiratory rate were monitored continuously during sessions. Sessions were stopped if patients had an abnormal physiologic response: HR > 70% of predicted maximum, >20% decrease in HR, SBP > 180 mm Hg, > 20% decrease in SBP or DBP, SpO_2_ < 90%, or clinical signs and symptoms of cardiorespiratory distress.

### Outcome measures

The primary outcome variables were levels of the circulating biomarkers: Klotho, FGF23, IL-6 and BDNF. The serum levels of FGF23, IL-6 and BDNF were determined using magnetic bead technology from LuminexTM with the Human Custom ProcartaPlex 3-plex (Affymetrix, Vienna, Austria) according to the manufacturer’s instructions. In addition, the levels of serum α-Klotho were determined using the Human soluble α-Klotho ELISA kit (Immuno-Biological Labs, Japan), following the manufacturer’s instructions, and analyzed by the Spark Microplate Reader (Tecan, Männedorf, Switzerland). The concentration of each factor was calculated from standard curves.

Secondary outcome variables were: (a) *Clinical (physical and cognitive function)*: bilateral tibialis anterior (TA) and quadriceps muscle strength measured using a hand-held dynamometer (Sauter, Kern, FK Digital dynamometer), Repeatable Battery for the Assessment of Neuropsychological Status (RBANS) test, Trail Making Test (TMT) and Mini-Mental State Examination (MMSE) scores; (b) *Neuroradiological*: structural and functional brain magnetic resonance imaging (MRI) features performed on a 3 T MRI scanner (Discovery 750w, General Electric Medical System) utilizing a 32-channel head coil. Participants were positioned in the scanner with their heads comfortably restrained by foam padding to reduce head movement. Earplugs were used to reduce the noise of the scanner.

Muscle strength and biomarker data were collected at baseline (before starting intervention, t_0_) and 3 months after hospital discharge (t_1_). Baseline brain MRI studies were performed at the earliest feasible time after enrolment, mostly within 2 days (mean 2.13 ± standard deviation 2.68) and at t_1_. Finally, cognitive assessments were performed only at t_1_. A more detailed description of the demographic and clinical data collected, serum analysis, muscle strength evaluation, cognitive testing and imaging analysis is provided in the Supplementary material.

### Data analysis

#### Sample size and power

We performed this pilot study to generate a formal sample size and power computations for planning a larger and more comprehensive prospective study.

#### Statistical analysis

Qualitative variables are described by frequency distributions and percentages, while quantitative variables are expressed by mean ± standard deviation (SD) or median ± interquartile range. To compare the two treatment groups, the chi-squared test and Fisher’s exact test were used for categorical variables, while the two-sample *t*-test and Wilcoxon-Mann–Whitney test were used for continuous variables when appropriate. Comparisons between two temporal observations of biomarkers were assessed with paired *t*-tests. Since the data were repeated, a generalized estimating equations approach was applied to describe the effect of treatment on biomarkers and on muscle strength. Moreover, a generalized linear model was used to describe the effect of education on cognitive assessment on the basis of treatment. *P*-values < 0.05 were considered statistically significant. All statistical analyses were performed using SAS software, version 9.4 (SAS Institute Inc., Cary, NC, United States).

## Results

The analyses included only patients who completed the entire protocol (55 patients in total, NMES group: 23; control group: 32). The subjects’ demographic and clinical data are reported in Table 1. The two groups (NMES and control) were homogenous in relation to baseline variables, length of stay (LOS) and number of rehabilitation sessions. None of the patients enrolled in the study experienced delirium during the hospital stay or perioperative stroke. Baseline muscle strength evaluations, biomarker values and structural brain MRI features are reported in Table 2.

**Table 1:** Demographic and clinical features of study participants.

	NMES group (*n* = 23)	Control group (*n* = 32)	*p* value
Age y	63.13 ± 8.67	62.13 ± 12.15	0.9591
Sex (M) n%	21 (91.30)	25 (78.13)	0.1925
Weight Kg	78.92 ± 11.79	77.44 ± 13.64	0.5277
BMI Kg/m^2^	27.66 ± 3.47	27.69 ± 4.75	0.7009
Education y	11.33 ± 4.42	10.33 ± 3.87	0.4352
Diabetes n%	9 (39.13)	13 (40.63)	0.9111
Chronic respiratory disease n%	2 (8.70)	6 (18.75)	0.2968
Chronic kidney disease n%	4 (17.39)	4 (12.50)	0.6118
Chronic liver disease n%	2 (9.09)	1 (3.13)	0.3470
Chronic cardiovascular disease n%	21 (91.30)	28 (87.50)	0.6553
Infection at admission n%	0 (0)	0 (0)	–
LOS (mean, days) (SD)	6.65 (4.75)	8.91 (11.61)	0.4141
Sessions, mean n (SD)	12.52 (7.42)	13.75 (7.72)	0.5494

The table shows that there are no statistically significant differences among demographic and clinical variables between the groups (NMES and control) at baseline.

The *p* value was obtained using the chi-squared test for qualitative variables and the *t*-test and Wilcoxon test for quantitative variables. LOS includes the time interval between the enrolment date and the hospital discharge date. Only patients hospitalized for less than 6 days were enrolled according to inclusion criteria. The number of sessions for the intervention group considers only the number of NMES sessions, but patients also underwent usual care sessions. Neuromuscular electrical stimulation (NMES); body mass index (BMI); length of stay (LOS); standard deviation (SD).

**Table 2:** Baseline muscle strength evaluations (Kg), biomarker values (pg/ml) and structural brain MRI features.

	NMES group (*n* = 23)	Control group (*n* = 32)	*p* value
Baseline muscle strength (Kg)			
Right tibial	80.95 (23.32)	91.58 (37.01)	0.2069
Left tibial	89.13 (31.97)	95.25 (39.46)	0.3648
Right quadriceps	106.53 (29.60)	111.58 (66.61)	0.8107
Left quadriceps	110.40 (35.79)	110.22 (62.98)	0.9486
Biomarker values (pg/ml)			
Klotho	380.66 (239.80)	399.89 (255.55)	0.8847
BDNF^†^	422.71 (678.02)	301.46 (532.15)	0.5956
FGF23^†^	6.03 (15.20)	10.72 (26.05)	0.5508
IL-6^†^	21.90 (19.93)	24.16 (36.44)	0.2119
Structural brain MRI features			
Microbleeds‡	4 (30.8%)	13 (68.4%)	**0.0361**
Acute lesions^§^	0 (0%)	4 (21.1%)	0.1021
Fazekas (grade 1)^¶^	10 (76.9%)	17 (94.4%)	0.2134
Fazekas (grade 2)^¶^	1 (7.7%)	1 (5.6%)	

Baseline muscle strength values are reported in kilograms (Kg) and standard deviation (SD) values are reported in brackets: there were no statistically significant differences between the NMES and control groups when the baseline muscle strength was evaluated with a dynamometer.

The mean values of 3 peak torque measurements in each muscle were considered. Biomarker values are reported in pg/ml with SD in brackets: no significant differences were found in the mean baseline values (+/− SD) of any biomarkers. Brain MRI: only 14/23 patients in the NMSE group and 23/32 patients in the control group agreed to undergo the imaging study. We did not find statistically significant differences in structural features in the baseline MRI studies (mainly exploring chronic acute cerebral vascular disease), except for microbleeds, which were significantly lower in the NMES group.

^†^BDNF, FGF23, IL-6 values were available for 21/23 patients in the NMES group and for 29/32 patients in the control group.

^‡^As for microbleeds, data were available for 13/14 patients in the NMES group and for 19/23 patients in the control group.

^§^Acute lesions were ischemic subclinical lesions evaluated in 11/14 patients in the NMES group and 19/23 in the control group.

^¶^The Fazekas score was computed for 13/14 individuals in the NMES group and for 18/23 individuals in the control group.

Kilogram (Kg); neuromuscular electrical stimulation (NMES); magnetic resonance imaging (MRI); brain-derived neurotrophic factor (BDNF); fibroblast growth factor 23 (FGF23); interleukin-6 (IL-6).

The primary outcome was the difference in biomarker values between the interventional and control groups 3 months after hospital discharge (mean values are reported in Table 3). There was no statistically significant difference between the two groups for Klotho, BDNF or FGF23, but IL-6 was significantly higher in the NMES group (Table 3).

**Table 3:** Biomarker values, cognitive assessment, muscle strength values and neuroimaging analysis 3 months after hospital discharge.

	NMES group (*n* = 23)	Control group (*n* = 32)	*p* value
Biomarker values (pg/ml)			
Klotho	396.03 (412.63)	314.55 (204.70)	0.6006
BDNF^†^	573.29 (733.71)	380.01 (569.66)	0.8749
FGF23^†^	8.89 (16.71)	8.00 (21.90)	0.1992
IL-6^†^	12.52 (15.12)	10.70 (37.44)	**0.0268**
Cognitive assessment			
MMSE	26.20 (1.62)	26.78 (1.90)	0.2896
Immediate memory	88.95 (20.28)	91.68 (16.03)	0.5272
Visuospatial ability	99.81 (20.77)	95.87 (14.28)	0.2874
Language	80.00 (13.59)	80.58 (6.73)	0.2532
Attention	86.45 (14.61)	88.90 (18.34)	0.6363
Delayed memory	95.00 (16.02)	92.26 (16.11)	0.5271
RBANS total score	87.95 (18.90)	84.71 (14.10)	0.6991
TMT-A^‡^	64.24 (68.36)	33.76 (17.16)	**0.0423**
TMT-B^‡^	182.09 (128.77)	116.60 (82.73)	**0.0203**
TMT-B-A^‡^	117.28 (83.80)	82.70 (74.86)	0.1058
PIQ^§^	106.61 (10.90)	106.96 (9.73)	0.9838
Muscle strength value†(Kg)			
Right tibial	108.73 (41.25)	104.72 (36.41)	0.7752
Left tibial	107.30 (54.60)	102.90 (36.60)	0.9214
Right quadriceps	141.63 (65.09)	132.89 (59.82)	0.7179
Left quadriceps	131.99 (68.44)	129.61 (54.92)	0.6732
Structural brain MRI features^¶^			
Microbleeds	4 (36.4%)	13 (68.4%)	0.0877
Acute lesions	0 (0%)	0 (0%)	-
Fazekas (grade 1)	10 (90.9%)	16 (94.1%)	0.7475
Fazekas (grade 2)	1 (9.1%)	1 (5.9%)	

Biomarker values assessed 3 months after hospital discharge (t_1_): statistically significant differences in IL-6 are in bold.

Cognitive assessment: mean scores and SD are reported for each test included in the RBANS (immediate memory, visuospatial abilities, language, attention, delayed memory) as well as total scores from the RBANS, MMSE and TMT. Also, premorbid intelligence quotient (PIQ) as a surrogate for cognitive evaluation before hospitalization was obtained. Muscle strength: values were obtained through a dynamometer. The mean values of 3 peak torque measurements in each muscle were considered. SD values are reported in brackets for each measurement. Structural neuroimaging: no statistically significant differences among the two treatment groups were reported at t_1_ for the structural features considered.

^†^BDNF/FGF23/IL-6 were evaluated in 30/32 patients in control group.

^‡^Mean values were computed on 15 out of 16 patients in the NMES group and in 21 out of 22 in the control group.

^§^Mean values were computed on 14 out of 16 patients in the NMES group and in 17 out of 22 in the control group.

^¶^Fazekas data were available for 11/23 patients in the NMES group and 17/32 in the control group. Microbleeds: data were available for 11/23 patients in the NMES group and 19/32 in the control group. Acute lesions: data were available for 10/23 patients in the NMES group and 19/32 in the control group.

Repeatable Battery for the Assessment of Neuropsychological Status (RBANS); Mini-Mental State Examination (MMSE); Trail Making Test (TMT); premorbid intelligence quotient (PIQ); standard deviation (SD); brain-derived neurotrophic factor (BDNF); fibroblast growth factor 23 (FGF23); interleukin-6 (IL-6); neuromuscular electrical stimulation (NMES).

Secondary outcome variables, the cognitive assessment performance scores obtained at MMSE, TMT A and B, and RBANS, are reported in Table 3. No significant differences were found in scores computed for any test exploring different cognitive functions. A statistically significant difference was reported in TMT B, a test of attention, with the control group displaying lower values (i.e., better attention). No statistically significant differences between either group was observed in muscle strength values or brain MRI structural features when evaluated 3 months after hospital discharge (Table 3).

Functional brain MRI imaging was fully acquired in 16 subjects (eight in each group). MRI exams not fully acquired or not suitable for image post-processing were excluded. The remaining patients eligible for MRI examination were not imaged due to lack of consent for imaging or the presence of metallic foreign bodies.

**Figure 1:**
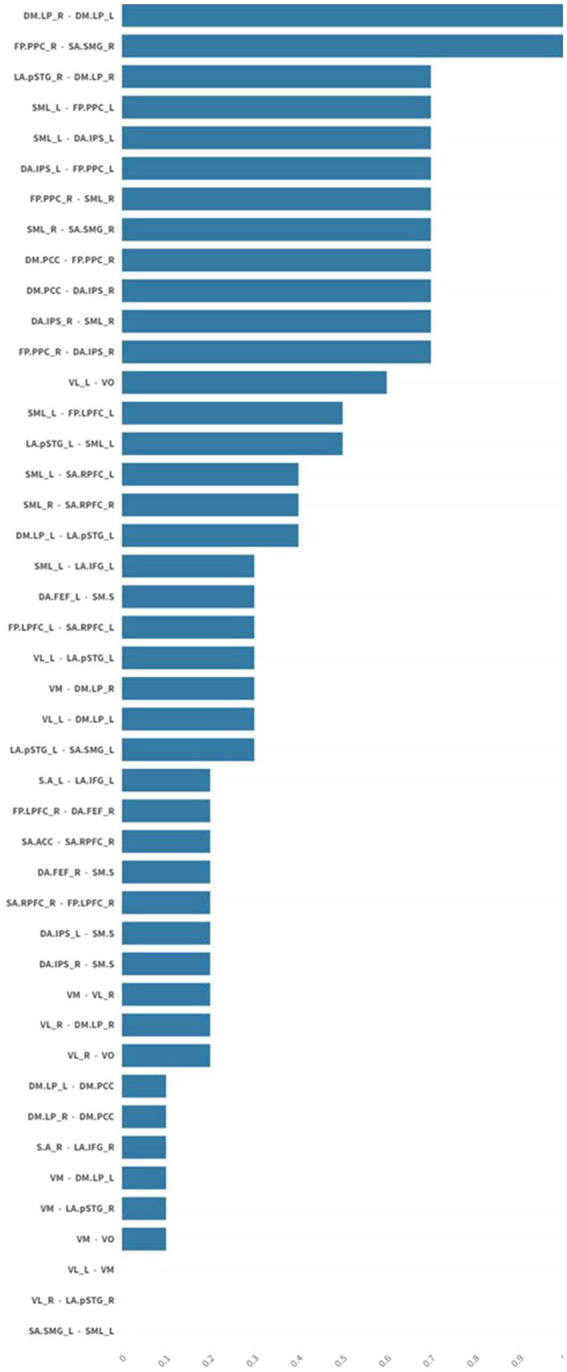
The graph shows mean effect size differences in resting-state network connectivity before and after usual care treatment. Mean effect sizes are represented as average normalized differences in Cohen’s d. There was a statistically significant (*p* 0.05 FDR) medium to large (Cohen’s *d* = 0.5 medium; Cohen’s *d* = 0.8 large) increase in functional connectivity in the default mode, frontoparietal, salience, and sensorimotor networks in patients after usual care treatment. False discovery rate (FDR).

**Figure 2:**
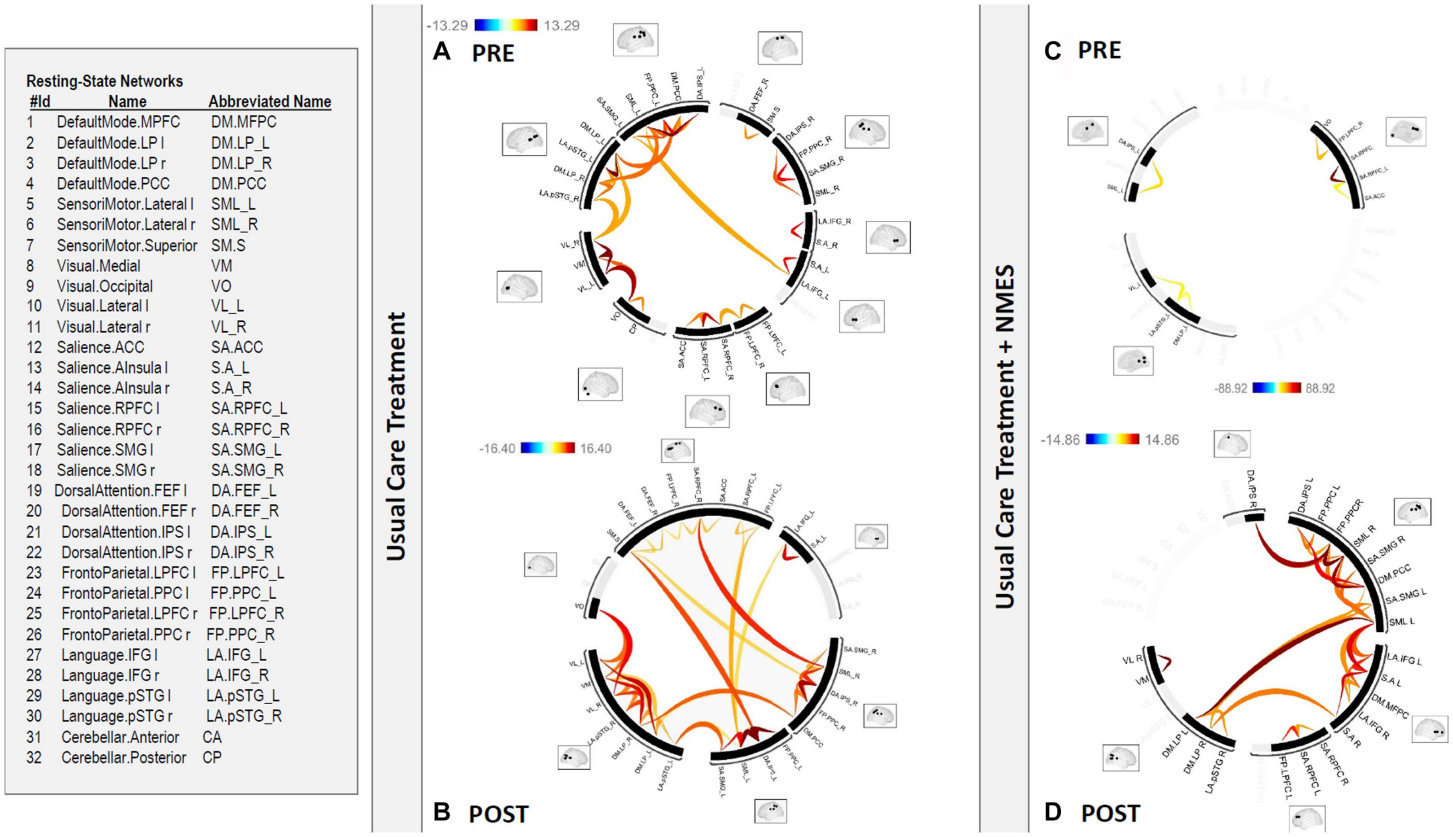
Functional connectome in patients **(A)** PRE and **(B)** POST usual care treatment versus **(C)** PRE and **(D)** POST NMES treatment. There was a statistically significant (*p* < 0.05 FDR) increase in functional connectivity in the default mode, frontoparietal, salience, and sensorimotor networks in patients after usual care treatment compared to pre-treatment functional connectivity in the same networks. This increased functional connectivity in the default mode, frontoparietal, salience, and sensorimotor networks was even greater for individuals in the usual care plus NMES group, compared to pre-treatment functional connectivity in the same networks, since the pre-treatment functional connectivity showed a prominent reduction in global functional connectivity in this group. Neuromuscular electrical stimulation (NMES); false discovery rate (FDR).

There was a statistically significant (*p* 0.05 false discovery rate (FDR)) medium to large (Cohen’s *d* = 0.5 medium; Cohen’s *d* = 0.8 large) increase in functional connectivity in the default mode, frontoparietal, salience, and sensorimotor networks in patients after usual care treatment compared to pre-treatment functional connectivity in the same networks (Figures 1, 2).

**Figure 3:**
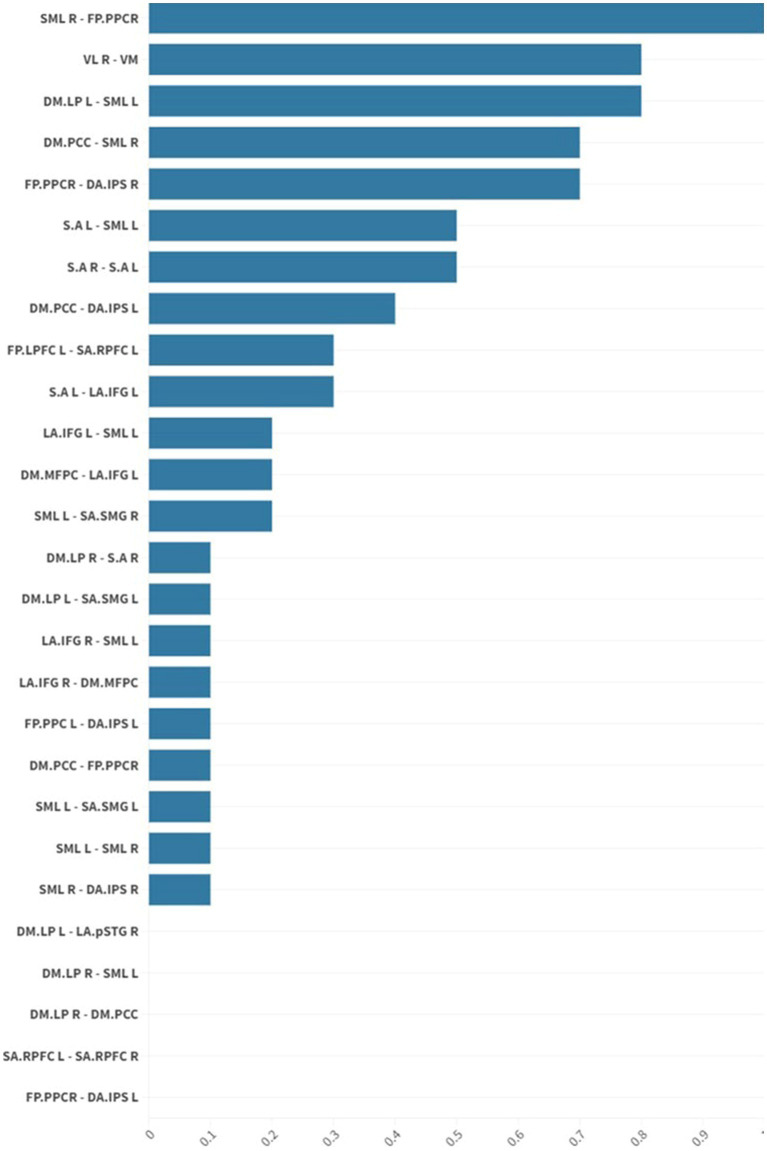
The graph shows mean effect size differences in resting-state network connectivity before and after intervention in the NMES group. Mean effect sizes are represented as average normalized differences in Cohen’s d. There was a statistically significant (*p* 0.05 FDR) medium to large (Cohen’s *d* = 0.5 medium; Cohen’s *d* = 0.8 large) increase in functional connectivity in the default mode, frontoparietal, salience, and sensorimotor networks for individuals in the usual care plus NMES treatment group. Neuromuscular electrical stimulation (NMES); false discovery rate (FDR).

There was a statistically significant (*p* 0.05 FDR) medium to large (Cohen’s *d* = 0.5 medium; Cohen’s *d* = 0.8 large) increase in functional connectivity in the default mode, frontoparietal, salience, and sensorimotor networks for individuals in the NMES group compared to pre-treatment functional connectivity in the same networks (Figures 2, 3). This finding was even more evident as the pre-treatment functional connectivity assessment showed a prominent reduction in global functional connectivity in this group.

Looking at the baseline muscle strength data, the NMES group seemed weaker than the control; for this reason, we performed an additional statistical analysis normalizing for baseline muscle strength, BMI and Klotho levels. There were no statistically significant differences in the primary outcome (Klotho levels at 3 months) between the two groups (NMES vs. usual care) (data not shown).

The above findings suggest the NMES protocol used in this study was likely to be insufficient to exert a significant additive effect over standard care alone on muscle and cognitive measures. For the next stage, we performed a supplementary analysis, stratifying patients according to the change (Δ) in Klotho (or other biomarker levels) from pre-to post-treatment [3 months after discharge (t_1_) relative to baseline assessment (t_0_)], regardless of the physical therapy protocol performed. Subjects were classified as “*responders*” (n°22 patients) if t_1_ Klotho values were increased compared to t_0_ assessment (Δ > 0; Δ range: 1.8–1099.32 pg/mL), and “*non-responders*” (n°31 patients) if follow-up values were decreased or unchanged from baseline (Δ ≤ 0; Δ range: −508.81 – −8.3). In Table 4, we report the demographic characteristics of both groups as well as the baseline muscle strength values and baseline biomarker values for responders and non-responders. We confirmed that the two groups of patients, responders and non-responders for Klotho, did not differ in most of the baseline clinical features, muscle strength or biomarker values. We only found that Klotho-responders had significantly lower baseline Klotho values than non-responders (*p* 0.002), a significantly lower percentage of diabetes (*p* 0.029) and finally that non-responders were predominantly male (*p* 0.0385).

**Table 4:** Demographic, clinical features and baseline physical and laboratory evaluations of responders and non-responders for Klotho.

	Responders (*n* = 22)	Non-responders (*n* = 31)	*p* value
Age y	62.64 ± 9.97	63.32 ± 11.64	0.7382
Gender (M) n%	18 (81.82)	26 (83.87)	**0.0385**
Weight Kg	78.00 ± 10.24	77.86 ± 14.80	0.8709
BMI Kg/m^2^	28.01 ± 3.09	27.68 ± 4.93	0.4482
Education y	10.71 ± 4.01	10.46 ± 4.17	0.8821
Diabetes n%	4 (18.18)	16 (51.61)	**0.0134**
Chronic respiratory disease n%	3 (13.64)	5 (16.13)	0.8028
Chronic kidney disease n%	2 (9.09)	6 (19.35)	0.3037
Chronic liver disease n%	2 (6.45)	1 (4.76)	0.7976
Chronic cardiovascular disease n%	20 (90.91)	27 (87.10)	0.6660
Infection at admission n%	0 (0)	0 (0)	–
LOS, n (mean days) (SD)^†^	7.62 (4.04)	8.00 (11.94)	0.8863
Muscle strength (Kg)			
Right tibial	76.52 (20.34)	94.30 (36.55)	0.1100
Left tibial	91.73 (29.64)	92.66 (38.59)	0.5909
Right quadriceps	104.60 (34.36)	106.20 (65.16)	0.2201
Left quadriceps	100.27 (31.10)	115.90 (65.17)	0.8722
Baseline biomarker values (pg/ml)			
Klotho	268.78 (252.03)	482.88 (202.27)	**0.0002**
BDNF	286.56 (518.45)	404.11 (652.28)	0.8222
FGF23	6.05 (17.43)	10.87 (25.25)	0.2518
IL-6	25.86 (40.64)	21.12 (19.47)	0.3843

Demographic and clinical features of responders and non-responders for Klotho are reported.

Klotho non-responders were predominantly men and more affected by diabetes (statistically significant *p* values in bold). Baseline muscle strength evaluations through dynamometer (in Kg) and biomarker values (pg/ml) are reported for Klotho responders and non-responders. No statistically significant differences were detected in baseline muscle strength in either patient group. Klotho responders showed lower baseline Klotho levels than non-responders (*p* value in bold).

^†^LOS for responders is computed in 21/22 patients since 1 outlier was removed from the analysis.

Male (M); body mass index (BMI); length of stay (LOS); kilogram (Kg); picogram per milliliter (pg/ml); brain-derived neurotrophic factor (BDNF); fibroblast growth factor 23 (FGF23); interleukin-6 (IL-6).

We performed the same classification (responders/non-responders) according to the other myokine trend values (data shown in Supplementary material, Results section: Supplementary Tables S2, S3); however, we mainly focused on Klotho, in consideration of the interesting findings reported below, which confirm the well-established neuroprotective role of this myokine (25, 26).

**Figure 4:**
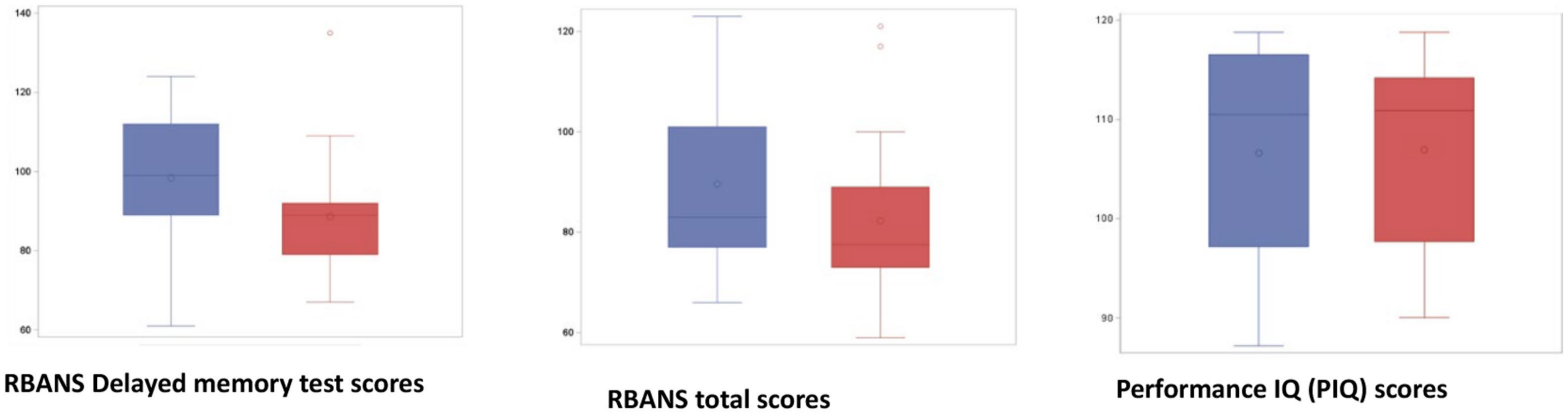
Partial and total RBANS scores in responders (blue) and non-responders for Klotho (red). Responders (blue) performed significantly better than non-responders (red) on the delayed memory test (*p* value = 0.02). A similar trend was also observed for all other cognitive tests included in RBANS without reaching significance. Performance of Premorbid Intelligence Quotient (PIQ) scores: The absence of differences between the PIQ of the two groups indicates that the differences in the NP tests are not attributable to different IQs. Repeatable Battery for the Assessment of Neuropsychological Status (RBANS); premorbid intelligence quotient (PIQ).

No significant differences were detected in cognitive performance between responders and non-responders when this classification was based on myokines other than Klotho (BDNF, FGF23, and IL-6 groups) (data shown in Supplementary material, Table S3). Klotho responders instead performed significantly better than non-responders on the delayed memory test (*p* 0.02). A similar trend was also observed for all the other cognitive tests included in RBANS, though the values did not reach statistical significance (Figure 4).

## Discussion

According to the results of this study, the addition of an NMES protocol to the normal standard of care during hospitalization only increased IL-6 levels when compared to standard care alone. Many biological roles have been described for muscle-derived IL-6, such as regulation of glucose homeostasis, regulation of fat metabolism, as well as positive immunological suppressive effects (27–29), but we did not find any direct impact on cognition consistent with our results. None of the other neuroprotective myokines were significantly different between groups. Nor were there differences between groups when considering the long-term cognitive function, muscle strength, or structural changes according to brain MRI of CABG patients. However, we did find that both groups yielded improvements in the functional connectome according to functional brain MRI, and NMES amplified these effects. Neuromuscular electrical stimulation (NMES) has been extensively adopted in rehabilitation settings as a complement and/or substitute for voluntary strength training when this is unfeasible (30). A positive role of NMES has been demonstrated in healthy seniors for increasing muscle size and strength, inducing molecular changes like upregulation of insulin-like growth factor (IGF-1) and modulation of the muscle RING-finger protein-1 (MuRF-1) in the skeletal muscle (31); upregulation of blood BDNF and Klotho in the hippocampus of normal rats has also been observed (32). Conversely, in our subjects NMES did not modulate secretion into the blood of potential neuroprotective myokines (Klotho, BDNF, FGF23), except for IL-6, whose pleiotropic functions in different tissues and organs have been described (33). In this human clinical setting, we cannot verify if there are some local effects in upregulation of Klotho expression in the hippocampus through an additional pathway of neurogenesis induced by the peripheral nervous system and activated by muscle stimulation (32). Since our protocol of NMES intervention did not produce any macroscopic changes in terms of cognitive performance, we can indirectly hypothesize that this intervention paradigm does not induce any significant local molecular changes in the brain either.

In the second part of this study, we stratified subjects according to those who demonstrated a positive response to intervention (“responders”), as determined by circulating biomarker levels, compared to those who did not demonstrate a biomarker response to intervention (“non-responders”), independent of the type of rehabilitation intervention (standard or standard + NMES). We found that, of all the biomarkers evaluated, only patients with a change in Klotho levels performed better on all cognitive tests, significantly on the delayed memory index of the RBANS test, which is a measure of delayed recall and recognition of verbal and visual information, indicating good functioning of the long-term memory store. This result supports, in a clinical research setting, the neuroprotective role of Klotho as a cognitive-enhancing protein (22). Increased endocrine function of the active muscle is one hypothesis to explain the anti-aging effects of physical activity (muscle-brain endocrine axis) (9), but it is still unknown whether skeletal muscle contractile activity itself results in the local production and secretion of Klotho into the bloodstream, or if some other myokine is responsible for inducing Klotho expression in other tissues, such as the brain. In murine and human models, exercise has been shown to be a potent stimulus for increased plasma Klotho levels, but that response may be dependent on physical fitness level as well as age (9). There must be additional factors, other than age and muscle strength, influencing Klotho expression or upregulation since we did not find any differences in those parameters between the two groups (responders and non-responders). According to our results, diabetes and male gender were more frequent in the non-responder group; therefore, metabolic factors, sex factors and other additional factors are worth exploring as triggers or modulators of Klotho expression. Also, baseline levels of Klotho, which may depend on gene polymorphism, influence post-operative and post-treatment Klotho levels.

It has been suggested that the Klotho protein may protect against cognitive decline in aging and neurodegenerative disorders through multiple mechanisms: such as promoting optimal synaptic function via activation of N-methyl-d-aspartate (NMDA) receptor signaling, stimulating the antioxidant defense system, reducing inflammation, promoting autophagy and enhancing clearance of amyloid-β (34). A number of epidemiological studies have associated elevated Klotho levels with better health outcomes and even extended life span in humans. Higher plasma Klotho concentrations were associated with lower risk of meaningful decline and a smaller average decline in the Mini Mental State Examination (MMSE), but not in executive function or motor skills tests (35). Zhao et al. showed that Klotho overexpression inhibited PYD domain-containing protein 3 (NLRP3) inflammasome activation and promoted Aβ clearance through an increase in M2 type microglia and the regulation of Aβ transporters in amyloid precursor protein/presenilin 1 (APP/PS1) mice, with effectively relieved neuroinflammation and Aβ burden and ameliorated Alzheimer’s Disease (AD)-like phenotypes (36). Like our findings, previous *in vivo* studies in mice showed that upregulation of Klotho improves aging-related memory deficits (37).

We recognize that our study has several limitations, mainly concerning the small sample size, the lack of cognitive assessment before surgery, and the limited time of follow-up. Since only a few brain MRI scans were obtained, we could not explore any plausible laboratory-clinical and imaging (anatomical and functional) correlations, nor could we explore functional neuroimaging differences between the two groups of Klotho responders and non-responders. Unfortunately, neither the timing (candidate for urgent surgery or in the post-operative status) nor the setting (hospitalization as in-patients) were appropriate for a baseline cognitive assessment. Many patients had contraindications to MRI scans or refused a time-consuming neuroimaging assessment for their post-operative status. On the other hand, we demonstrated a cognition enhancing role of Klotho in a surgical setting with the potential to reverse, or at least reduce, post-operative cognitive decline after cardiac surgery. We can further speculate that having lower pre-operative plasma levels of Klotho can be a biomarker of lower post-operative values, potentially associated with worse memory performances. We still need to understand which factors induce Klotho expression, particularly modifiable factors, like a specific rehabilitation program, in order to design appropriate exercise protocols to counteract the deleterious effects of surgery, above all in high-risk populations (CABG candidates with low baseline Klotho levels). In addition, it is worth investigating in future studies whether specific cognitive function improvement is associated with concomitant anatomical or functional changes in specific brain regions.

The main achievement of our study is the confirmation, in a clinical setting, that Klotho has potential neuroprotective effects, preserving or improving memory function not only in physiological aging but also in this post-operative setting characterized by an increased risk of cognitive impairment.

It paves the way for further research questions, for example, relating to how an exercise program and factors other than physical exercise, for instance sex and age, influence the endocrine function of skeletal muscle, or testing the potential treatment application of Klotho as a drug in this patient category.

## Data availability statement

The raw data supporting the conclusions of this article will be made available by the authors, without undue reservation.

## Ethics statement

The studies involving humans were approved by Comitato Etico IRCCS Sicilia-Sezione ISMETT IRCCS srl. The studies were conducted in accordance with the local legislation and institutional requirements. The participants provided their written informed consent to participate in this study.

## Author contributions

VL and FAm developed the ideas in the study, defined goals and endpoints, conceived the study design, and drafted the manuscript. GRu coordinated the research activities and data management. EL, MB, GI, DT, AS, FAv, GS, GeM, GPan, GRa, and MP collected the data and participated in data acquisition and interpretation of results. RA and FT contributed to the study methods and performed the data analysis. SA contributed with data collection and Tables/Figures drafting. GPar contributed with analysis and explanation of functional imaging. GV supported with the acquisition of funding and writing. PC was responsible for and supervised all research activities and edited the manuscript. All authors participated in drafting the manuscript sections and reviewing them critically, contributed to scientific and intellectual content, and approved the final manuscript.

## Funding

This work was supported by the Italian Ministry of Economic Development (Horizon 2020 PON I&C 2014-2020, Lifestyle4Health F/050408/01-03/X32) and partially funded by the Italian Health Ministry, Ricerca Corrente 2023.

## Conflict of interest

The authors declare that the research was conducted in the absence of any commercial or financial relationships that could be construed as a potential conflict of interest.

## Publisher’s note

All claims expressed in this article are solely those of the authors and do not necessarily represent those of their affiliated organizations, or those of the publisher, the editors and the reviewers. Any product that may be evaluated in this article, or claim that may be made by its manufacturer, is not guaranteed or endorsed by the publisher.

## Footnotes

^1^ https://projectredcap.org/

## Glossary

coronary artery bypass grafting: CABG
blood–brain barrier: BBB
post-operative cognitive decline: POCD
intensive care unit: ICU
brain-derived neurotrophic factor: BDNF
fibroblast growth factor 23: FGF23
interleukin-6: IL-6
neuromuscular electrical stimulation: NMES
heart failure: HF
critical coronary artery disease: CAD
cardiothoracic unit: CTU
Brief Intelligence Test: TIB
hertz: Hz
microseconds: μs
revolutions per minute: rpm
heart rate: HR
systolic blood pressure: SBP
diastolic blood pressure: DBP
saturation of peripheral oxygen: SpO_2_
magnetic resonance imaging: MRI
Repeatable Battery for the Assessment of Neuropsychological Status: RBANS
Mini-Mental State Examination: MMSE
body mass index: BMI
chronic obstructive pulmonary disease: COPD
tibialis anterior: TA
maximum voluntary push torque: MVPT
Trail Making Test: TMT
fast spin-echo: FSE
fluid-attenuated inversion recovery: FLAIR
gradient-recalled-echo: GRE
susceptibility weighted imaging: SWI
arterial spin labeling: ASL
cerebral blood flow: CBF
dynamic susceptibility perfusion weighted imaging: DSC-PWI
cerebral blood volume: rCBV
cerebral blood flow: rCBF
mean transit time: MTT
resting-state functional MR imaging: rest-fMRI
blood oxygenation level dependent: BOLD
default mode network: DMN
Montreal Neurological Institute: MNI
Statistical Parametric Mapping-12: SPM12
generalized estimating equations: GEE
generalized linear model: GLM
false discovery rate: FDR
standard deviation: SD
premorbid intelligence quotient: PIQ
length of stay: LOS
physical therapy: PT
PYD domain-containing protein 3: NLRP3
amyloid precursor protein/presenilin 1: APP/PS1
Alzheimer’s Disease: AD
insulin-like growth factor: IGF-1
muscle RING-finger protein-1: MuRF-1
