# Supplementary material for: Cognitive outcomes in patients treated with neuromuscular electrical stimulation after coronary artery bypass grafting
Source: Front Neurol. 2023 Aug 25;14:1209905. doi: 10.3389/fneur.2023.1209905 (PMC10486105; doi:10.3389/fneur.2023.1209905)
Supplement: Supplementary file 1 [file Data_Sheet_1.docx]

**SUPPLEMENTARY MATERIALS**

**Methods**

Demographic and clinical data: Data on age, sex, education, weight, body mass index (BMI), chronic obstructive pulmonary disease (COPD), diabetes mellitus, chronic kidney disease, chronic hepatic disease, infectious disease at admission. length of stay (LOS), type of admission was collected.

Serum analysis: A serum blood draw was obtained at baseline (pre-intervention) and 3 months after discharge. Soluble Klotho, BDNF, FGF23 and IL-6 were quantified using sandwich enzyme-linked immunosorbent assays and compared across intervention groups. All values are reported in pg/ml.

Muscle strength: Muscle strength testing was performed at baseline, during the hospital stay after treatment (only when feasible) and 3 months after discharge by a trained clinician. Tibialis anterior (TA) and quadriceps muscle strength were measured using a hand-held dynamometer (Sauter, Kern, FK Digital dynamometer). For quadriceps muscle strength, the maximum voluntary push torque (MVPT) for knee extension was tested at a knee angle of 25 degrees. An angle of 25 degrees was selected to correspond to the knee angle at which force production is of crucial importance in walking. Patients were positioned sitting upright, with no back support, and with hips at 90 degree flexion. The thigh of the patient was stabilized by the examiner's hand. The dynamometer was positioned perpendicular to the tibia, at 80% of the shank length, between the marks at the lower edge of the lateral epicondyle and the lower edge of the lateral malleolus, distal to the knee. The knee joint center and the 80% shank length were marked with a dot on the patients' skin. Strength of the TA was tested in a similar position, with the ankle positioned at 90 degrees, and the hand-held dynamometer placed on the dorsum of the foot. The patient was asked to exert a maximal isometric contraction while the examiner held the dynamometer in a fixed position. The average of 3 peak torque measurements and the highest value of the 3 peak torque measurements were recorded. All values are reported in kilograms (Kg).

Cognitive testing: A neuropsychologist assessed the subjects’ global cognition and executive function using the RBANS, TMT and MMSE. A baseline pre-surgery cognitive assessment was not performed since patients were hospitalized immediately before surgery. The RBANS is composed of 12 subtests and generates scores in five cognitive domains (immediate memory, attention, language, visuospatial/constructional ability and delayed memory) and a global index score. Patients’ scores were converted to standardized values (i.e. index scores), in which the normative range is a mean of 100 ± 15 (1 SD), and a score < 75 indicates a pathological performance [Randolph C et al., 1998; Wood MD et al., 2018]. The TMT is a neuropsychological test extensively used to evaluate attention, visual search, processing speed, task switching, cognitive flexibility and executive function [Talwar N et al., 2020]. In order to exclude pre-existing cognitive impairments, the Brief Intelligence Test (TIB) was also administered. The TIB [Colombo L et al., 2002] provides an estimate of premorbid intelligence by reading a list of regular and irregular Italian words. Scores <93.1 at TIB indicate a pathological performance.

Imaging analysis:

MRI examinations were performed on a 3T MRI scanner (Discovery 750w, General Electric Medical System) utilizing a 32-channel head coil. Participants were positioned in the scanner with their heads comfortably restrained by foam padding to reduce head movement. Earplugs were used to reduce the noise of the scanner.

During the resting-state scans, participants were instructed to keep their eyes closed, remain as motionless as possible and were asked not to think about any specific thoughts and not to count or perform any specific, rhythmic mental activity. Standard multi-parametric MR imaging protocol: MR imaging with fast spin- echo (FSE) T1-weighted and T2-weighted MR images, fluid-attenuated inversion recovery (FLAIR), T2*-weighted gradient-recalled-echo (GRE), susceptibility weighted imaging (SWI) and standard 3-direction diffusion weighted imaging (b=0, 1000) were obtained.

MRI examinations included the standard MRI protocol as listed above, plus isotropic T1-weighted volumetric imaging (3D-SPGR or MPRAGE) to assess cortical grey matter volume changes.

Functional MRI: resting-state functional MR imaging (rest-fMRI) with blood oxygenation level dependent (BOLD) echo-planar imaging technique was performed to assess spontaneous neuronal activity within the resting-state networks (RSN) to assess cerebral network connectivity. There were 210 volumes in total.

Imaging finding assessment: MR images were evaluated for the presence of acute ischemic brain lesions subdivided and categorized by anatomic brain location (basal ganglia, frontal lobes, parietal lobes, occipital lobes). The number, size and distribution of the lesions were recorded.

Susceptibility-weighted images were evaluated to assess the presence of hemorrhagic components. Advanced MRI: Voxel-based morphometry analysis was conducted after co-registration to a standardized brain template (Montreal Neurological Institute: MNI https://brainmap.org/training/BrettTransform.html) to assess cortical gray matter volume changes using the computational anatomy toolbox (CAT12) implemented in Statistical Parametric Mapping 12 (SPM12) (http://www.fil.ion.ucl.ac.uk/spm), a suite of MATLAB (https://www.mathworks.com/).

Brain connectivity network analyses were conducted using resting-state functional MR images. To assess functional connectivity, the CONN toolbox (https://web.conn-toolbox.org/) was used. CONN toolbox (https://www.nitrc.org/projects/conn) is an open-source MATLAB/SPM-based cross-platform software for the computation, display, and analysis of functional connectivity.

Data pre-processing: SPM 12 and CONN toolbox [Nieto-Castanon A et al., 2022] were used for functional image preprocessing [Nieto-Castanon A et al., 2022]. The first 10 volumes of the functional time series were discarded for signal stabilization.

Registration of the first volume was performed to correct for head movement. Rotation and movement of all participants was within 2mm in the x, y, and z planes. Volumes were normalized to the standard EPI template in MNI space and restored to 3 × 3 × 3 mm^3^. The resulting images were spatially smoothed with an 8mm full-width at half-maximum Gaussian kernel. Subsequently, the CompCor function was used for spatial and temporal preprocessing to minimize the impact of motion and physiological noise factors, as well as to define and remove confounds in the BOLD signal [Behzadi Y et al., 2007]. Regression of first order derivative terms for the whole brain, ventricular, and white matter signals were also included in the correlation preprocessing. This regression process was used to reduce the influence of spurious variance on neuronal activity.

Seed selection and functional connectivity analysis: assessment of the functional connectivity matrix in selected seed areas in the resting-state networks was carried out by an ROI-to-ROI connectivity matrix comparison and by testing hypotheses. Data visualization was obtained using the CONN toolbox and SPM12. All ROIs were imported into the CONN Toolbox. Functional connectivity measures were computed between seed areas for ROI-to-ROI analysis and to identify patterns of ROI-to-ROI connectivity. The CONN toolbox was used to obtain a linear measure of functional connectivity based on bivariate correlation and bivariate regression coefficients and their associated multivariate measures of semi-partial correlation and multivariate regression coefficients. Functional connectivity between the two groups at baseline and follow-up was assessed using two-tailed paired t-tests. The resulting statistical maps were set with p < 0.05 at the cluster level, false discovery rate (FDR) corrected. For maps that did not meet this criterion, a p < 0.001 threshold level was used.

Behavioral correlations: to investigate the changes in functional connectivity before and after the two treatments (usual care vs usual care plus NMES), we conducted a correlation analysis based on graph theory-based methods. Data were deemed to be statistically significant if pFDR−corrected < 0.05 (Benjamini and Hochberg FDR). Data are presented as mean effect size differences in resting-state network connectivity before and after each treatment. Mean effect sizes are represented as average normalized differences in Cohen's d, computed from t-statistic and degrees of freedom (DOFs) as d = T / sqrt(dof).

Thus, the effect size is a value measuring the degree of change in functional connectivity before and after each treatment. Cohen’s d effect size interpretation is as follows: 0.2 small; 0.5 medium; 0.8 large.

Table S1 presents resting-state network names and Montreal Neurological Institute (MNI) coordinates.

**Table S1.** Resting-state network names and Montreal Neurological Institute (MNI) coordinates


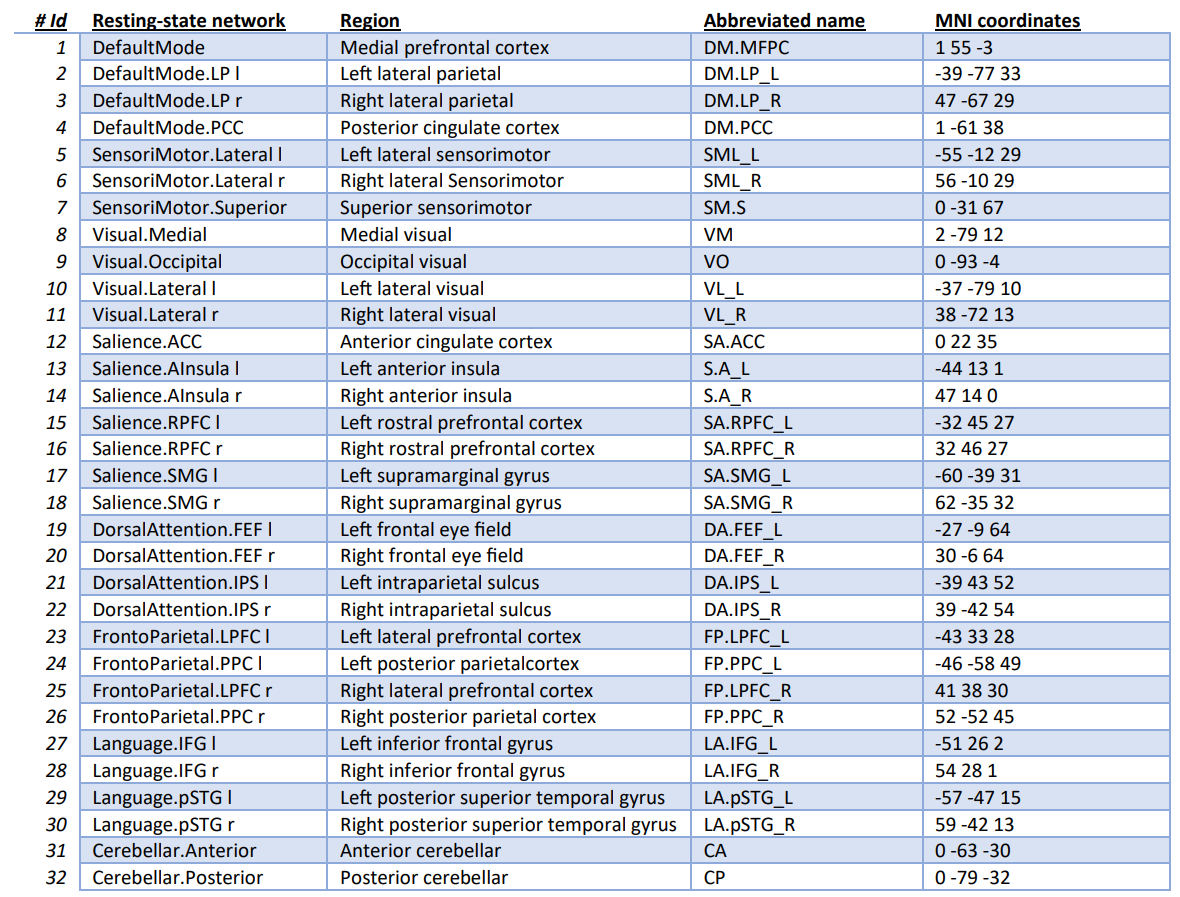


**Results**

**Table S2.** Demographics, clinical features, baseline muscle strength and baseline myokine values in *responders* and *non-responders* (Δ myokine values t_1_ – t_0_) for BDNF, FGF23 and IL-6

|  | BDNF | | FGF23 | | IL-6 | |
| --- | --- | --- | --- | --- | --- | --- |
|  | **Non-Responders (n= 29)** | **Responders (n= 22)** | **Non-Responders (n=8)** | **Responders (n= 25)** | **Non-Responders (n= 38)** | **Responders (n=9)** |
| Age | 61.21 ± 12.43 | 64.04 ± 8.76 | 62.38 ± 11.87 | 69.8 ± 8.93 | 63.82 ± 11.46 | 61.78 ± 7.03 |
| Weight | 75.32 ± 13.22 | 81.05 ± 12.23 | 82.86 ± 18.75 | 73.5 ± 13.3 | 76.59 ± 12.01 | 78.18 ± 12.35 |
| BMI | 27.41 ± 4.52 | 28.32 ± 3.88 | 27.75 ± 5.53 | 26.36 ± 4.6 | 27.94 ± 4.2 | 26.28 ± 3.39 |
| Education | 9.88 ± 3.75 | 11.35 ± 4.34 | 12.17 ± 4.17 | 12.5 ± 3.7 | 10.46 ± 4.17 | 11.14 ± 4.26 |
| Gender (male) | 23 (79%) | 21 (88%) | 7 (88%) | 4 (80%) | 31 (82%) | 8 (89%) |
| Diabetes | 13 (45%) | 7 (29%) | 2 (25%) | 2 (40%) | 13 (34%) | 4 (44%) |
| Chronic respiratory disease | 6 (21%) | 2 (8%) | 2 (25%) | 0 (0%) | 7 (18%) | 0 (0%) |
| Chronic cardiovascular disease | 25 (86%) | 22 (92%) | 8 (100%) | 5 (100%) | 34 (89%) | 7 (78%) |
| Chronic kidney disease | 5 (17%) | 3 (12%) | 2 (25%) | 0 (0%) | 5 (13%) | 2 (22%) |
| Chronic liver disease | 2 (7%) | 1 (4%) | 0 (0%) | 1 (20%) | 1 (3%) | 2 (22%) |
| Infection and admission | 0 (0%) | 0 (0%) | 0 (0%) | 0 (0%) | 0 (0%) | 0 (0%) |
| Left Quadriceps | 106.4 ± 60.64 | 115.29 ± 41.79 | 128.14 ± 71.33 | 120.3 ± 33.75 | 99.07 ± 34.35 | 148.59 ± 94.49 |
| Right Quadriceps | 107.25 ± 62.97 | 112.81 ± 41.55 | 126.79 ± 55.84 | 124 ± 34.25 | 101.43 ± 37.6 | 129.66 ± 95.58 |
| Left Tibial | 89.79 ± 38.79 | 95.91 ± 34.83 | 87.16 ± 41.6 | 82.34 ± 19.36 | 85.71 ± 28.96 | 114.64 ± 57.77 |
| Right Tibial | 82.57 ± 35.16 | 91.57 ± 28.63 | 85.89 ± 41.05 | 85.28 ± 13.63 | 80.5 ± 27.36 | 105.83 ± 43.82 |
| Klotho | 424.25 ± 258.38 | 357.47 ± 230.61 | 230.15 ± 205.77 | 366.25 ± 194.35 | 414.9 ± 263.97 | 417.18 ± 164.73 |
| BDNF | 445.45 ± 651.1 | 243.14 ± 512.21 | 74.67 ± 91.78 | 41 ± 18.63 | 400.47 ± 634.88 | 204.2 ± 500.84 |
| FGF23 | 7.36 ± 19.68 | 10.38 ± 24.95 | **51.59 ± 29.79** | **4.95 ± 4.52** | 7.02 ± 20.18 | 3.67 ± 5.99 |
| IL-6 | 29.61 ± 37.14 | 15.69 ± 17.74 | 12.61 ± 19.72 | 38.66 ± 32.06 | 27.23 ± 33.24 | 13.76 ± 12.96 |

**Table S2.** Demographic and clinical data are shown, as well as baseline muscle strength and biomarker values, for *responders* and *non-responders* according to myokine (other than Klotho) difference values between t_1_ and t_0_. No statistically significant differences were found among the groups, except for FGF23 values in the FGF23 groups: baseline values will influence the responder/non-responder status at t_1_ (values are reported in bold; *p =0.003*).

brain-derived neurotrophic factor (BDNF); fibroblast growth factor 23 (FGF23); interleukin-6 (IL-6); body mass index (BMI).

**Table S3.** Mean and standard deviation of scores of cognitive test in *responders* and *non-responders* (Δ myokine values t_1_ – t_0_) for Klotho, BDNF, FGF23 and IL-6.

|  | Klotho |  | | BDNF | | | FGF23 | | IL-6 | |
| --- | --- | --- | --- | --- | --- | --- | --- | --- | --- | --- |
|  | **Non-Responders**  **(n=31)** | | **Responders**  **(n=29)** | | **Non-Responders**  **(n= 29)** | **Responders**  **(n= 22)** | **Non-Responders**  **(n=8)** | **Responders**  **(n= 25)** | **Non-Responders (n= 38)** | **Responders (n=9)** |
| Immediate Memory | 87.14 ± 19.06 | 94.05 ± 15.54 | | 89.74 ± 19.47 | | 90.54 ± 16.12 | 88.14 ± 19.17 | 91.6 ± 34.67 | 88.7 ± 18.28 | 91.88 ± 17.23 |
| Visuospatial Ability | 94.34 ± 17.65 | 100.29 ± 15.93 | | 93.74 ± 14.4 | | 100.48 ± 19.4 | 104 ± 11.71 | 91.4 ± 25.07 | 94.89 ± 13.94 | 103.75 ± 27.04 |
| Language | 78.41 ± 9.55 | 82.82 ± 10.79 | | 78.85 ± 9.07 | | 81.96 ± 11.38 | 83.14 ± 11.74 | 84 ± 22.49 | 79.65 ± 10.04 | 85.62 ± 10.27 |
| Attention | 87.07 ± 16.2 | 88.29 ± 18.47 | | 87.38 ± 19.51 | | 87.83 ± 14.15 | 89.5 ± 14.71 | 86 ± 20.38 | 87.6 ± 18.31 | 87.5 ± 15.33 |
| Delayed Memory | **88.66 ± 13.51** | **98.36 ± 17.6** | | 92 ± 15.56 | | 93.79 ± 16.76 | 90.43 ± 20.82 | 93 ± 12.63 | 91.59 ± 13.91 | 96.25 ± 20.44 |
| RBANS Total Score | 82.29 ± 14.56 | 89.62 ± 17.33 | | 84.27 ± 14.37 | | 86.74 ± 18.02 | 88.17 ± 17.76 | 88.8 ± 24.65 | 83.74 ± 14.76 | 91.25 ± 20.53 |
| PIQ | 104.9 ± 10.45 | 108.54 ± 9.61 | | 104.9 ± 10.45 | | 107.48 ± 10.69 | 109.92 ± 10.42 | 105.11 ± 13.95 | 106.56 ± 10.24 | 108.72 ± 10.32 |
| TMT-A | 49.02 ± 61.27 | 43.94 ± 25.29 | | 48.09 ± 58.84 | | 45.56 ± 37.23 | 33.96 ± 11.5 | 70.8 ± 73.69 | 47.72 ± 51.86 | 48.23 ± 54.47 |
| TMT-B | 144.85 ± 106.82 | 149.72 ± 114.79 | | 141.4 ± 118.66 | | 152.5 ± 101 | 158 ± 78.19 | 123.25 ± 63.03 | 164.33 ± 122.54 | 105 ± 56.81 |
| TMT (B-A) | 95.96 ± 68.25 | 104.84 ± 95.19 | | 94.02 ± 73.4 | | 105.57 ± 87.6 | 126 ± 86.81 | 52.5 ± 14.75 | 116.12 ± 88.57 | 56.75 ± 30.48 |
| MMSE | 26.42 ± 1.74 | 26.67 ± 1.72 | | 26.65 ± 1.89 | | 26.39 ± 1.54 | 26.6 ± 1.64 | 26.06 ± 2.39 | 26.61 ± 1.69 | 25.9 ± 1.98 |

**Table S3.** Immediate memory, visuospatial ability, language, attention, and delayed memory are sub scales of the RBANS battery. A statistically significant difference was found only in the delayed memory score between Klotho responders and non-responders as highlighted in bold (*p=0.02*).

brain-derived neurotrophic factor (BDNF); fibroblast growth factor 23 (FGF23); interleukin-6 (IL-6); Repeatable Battery for the Assessment of Neuropsychological Status (RBANS); Mini-Mental State Examination (MMSE); Trail Making Test (TMT); premorbid intelligence quotient (PIQ)
